# Towards harmonisation of entomological surveillance in the Mediterranean area

**DOI:** 10.1371/journal.pntd.0007314

**Published:** 2019-06-13

**Authors:** Frédéric Jourdain, Abdallah M. Samy, Afrim Hamidi, Ali Bouattour, Bülent Alten, Chafika Faraj, David Roiz, Dušan Petrić, Elisa Pérez-Ramírez, Enkeledja Velo, Filiz Günay, Golubinka Bosevska, Ibrahim Salem, Igor Pajovic, Jelena Marić, Khalil Kanani, Lusine Paronyan, Maria-Grazia Dente, Marie Picard, Marija Zgomba, M'hammed Sarih, Nabil Haddad, Oleksandr Gaidash, Roena Sukhiasvili, Silvia Declich, Taher Shaibi, Tatiana Sulesco, Zoubir Harrat, Vincent Robert

**Affiliations:** 1 French National Research Institute for Sustainable Development, Research unit MIVEGC IRD-CNRS-Montpellier University, Montpellier, France; 2 Entomology Department, Faculty of Science, Ain Shams University, Cairo, Egypt; 3 University of Prishtina, Faculty of Agriculture and Veterinary Sciences, Prishtina, Kosovo; 4 Université de Tunis El Manar, Institut Pasteur de Tunis, LR11IPT03 Service d’entomologie médicale, Tunis, Tunisia; 5 Hacettepe University, Faculty of Science, Biology Department, Ecology Section, Ankara, Turkey; 6 Laboratoire d'Entomologie Médicale, Institut National d'Hygiène, Rabat, Morocco; 7 Faculty of Agriculture, Department of Phytomedicine and Environment Protection, Laboratory for Medical Entomology, University of Novi Sad, Novi Sad, Serbia; 8 Centro de Investigación en Sanidad Animal, Instituto Nacional de Investigación y Tecnología Agraria y Alimentaria (INIA-CISA), Carretera Algete-El Casar, Valdeolmos, Madrid, Spain; 9 Control of Infectious Diseases Department, Institute of Public Health, Tirana, Albania; 10 Institute of Public Health of R. Macedonia, Laboratory for virology and molecular diagnostics, Skopje, the Former Yugoslav Republic of Macedonia; 11 Ministry of Health, Central public health laboratory, Ramallah, Palestine; 12 University of Montenegro, Biotechnical Faculty, Podgorica, Montenegro; 13 PI Veterinary Institute of the Republic of Srpska, Banja Luka, Bosnia and Herzegovina; 14 Parasitic and Zoonotic Diseases Department, Vector-Borne Diseases programmes manager, MOH, Ramallah, Jordan; 15 Epidemiology of Vector borne and Parasitic diseases, National Center for Disease Control and Prevention, Ministry of Health, Yerevan, Armenia; 16 National Center for Global Health, Istituto Superiore di Sanità, Rome, Italy; 17 Laboratoire des Maladies Vectorielles, Institut Pasteur du Maroc, Casablanca, Morocco; 18 Laboratory of Immunology and Vector-Borne Diseases, Faculty of Public Health, Lebanese University, Fanar, Lebanon; 19 State Body “Ukrainian I. I. Mechnikov Research Anti-Plague Institute of Ministry of Health of Ukraine”, Laboratory of Especially Dangerous Infections Epizootology, Odessa, Ukraine; 20 National Center for Disease Control and Public Health, Tbilisi, Georgia; 21 Reference Laboratory of Parasites & Vector Borne Diseases, NCDC Libya, and Zoology Department, Faculty of Science, University of Tripoli, Libya; 22 Institute of Zoology, Ministry of Education, Culture and Research, Chisinau, Moldova; 23 Laboratoire éco-épidémiologie Parasitaire et Génétique des Populations, Institut Pasteur d’Algérie, Algiers, Algeria; London School of Hygiene & Tropical Medicine, UNITED KINGDOM

## Abstract

**Background:**

The Mediterranean Basin is historically a hotspot for trade, transport, and migration. As a result, countries surrounding the Mediterranean Sea share common public health threats. Among them are vector-borne diseases, and in particular, mosquito-borne viral diseases are prime candidates as (re)emerging diseases and are likely to spread across the area. Improving preparedness and response capacities to these threats at the regional level is therefore a major issue.

The implementation of entomological surveillance is, in particular, of utmost importance. Guidance in designing entomological surveillance systems is critical, and these systems may pursue different specific objectives depending on the disease.

The purpose of the proposed review is to draw up guidelines for designing effective and sustainable entomological surveillance systems in order to improve preparedness and response. However, we make it clear that there is no universal surveillance system, so the thinking behind harmonisation is to define evidence-based standards in order to promote best practises, identify the most appropriate surveillance activities, and optimise the use of resources.

Such guidance is aimed at policymakers and diverse stakeholders and is intended to be used as a framework for the implementation of entomological surveillance programmes. It will also be useful to collaborate and share information with health professionals involved in other areas of disease surveillance. Medical entomologists and vector control professionals will be able to refer to this report to advocate for tailored entomological surveillance strategies.

The main threats targeted in this review are the vectors of dengue virus, chikungunya virus, Zika virus, West Nile virus, and Rift Valley fever virus. The vectors of all these arboviruses are mosquitoes.

**Methods:**

Current knowledge on vector surveillance in the Mediterranean area is reviewed. The analysis was carried out by a collaboration of the medical entomology experts in the region, all of whom belong to the MediLabSecure network, which is currently funded by the European Union and represents an international effort encompassing 19 countries in the Mediterranean and Black Sea region.

**Findings:**

Robust surveillance systems are required to address the globalisation of emerging arboviruses. The prevention and management of mosquito-borne viral diseases must be addressed in the prism of a One Health strategy that includes entomological surveillance as an integral part of the policy. Entomological surveillance systems should be designed according to the entomological and epidemiological context and must have well-defined objectives in order to effect a tailored and graduated response. We therefore rely on different scenarios according to different entomological and epidemiological contexts and set out detailed objectives of surveillance. The development of multidisciplinary networks involving both academics and public authorities will provide resources to address these health challenges by promoting good practises in surveillance (identification of surveillance aims, design of surveillance systems, data collection, dissemination of surveillance results, evaluation of surveillance activities) and through the sharing of effective knowledge and information. These networks will also contribute to capacity building and stronger collaborations between sectors at both the local and regional levels. Finally, concrete guidance is offered on the vector of the main arbovirus based on the current situation in the area.

## Introduction

The incidence and geographical distribution of arboviruses, particularly those transmitted by mosquitoes, are on the rise [1]. Different factors concur with this upsurge, including growing trends in global travel, trade, urbanisation and tourism, climate change, and land use changes and management [1,2]. In other words, the impact of arboviruses on public health is worsening, and there is no reason to expect any spontaneous improvement in the short term [1,3].

MediLabSecure is a European project aimed at improving the surveillance and monitoring of mosquito-borne viral diseases [4]. Within this framework, we offer guidance for harmonising entomological surveillance around the Mediterranean area by establishing common, evidence-based standards to promote best practises and identify the most appropriate surveillance activities while optimising financial and human resources. This approach is consistent with the One Health concept, defined by the One Health Initiative (http://www.onehealthinitiative.com) as ‘a worldwide strategy for expanding interdisciplinary collaborations and communications in all aspects of healthcare for humans, animals and the environment’. The surveillance of vector-borne diseases (VBDs) needs, therefore, to integrate the various components that determine the occurrence of these diseases into a system that can exploit relevant sources of data to deal with these risks. Ideally, the system will compile data from human surveillance (human cases), veterinary surveillance (animal hosts), entomological surveillance (arthropod vectors), and environmental surveillance (environmental risk factors) [5].

It is intended that the guidance be used by policymakers and diverse stakeholders as a framework when establishing and implementing entomological surveillance programmes. It might also be usefully shared with health professionals involved in other areas of disease surveillance, thus providing a common framework and raising awareness of the possibilities and limitations of entomological surveillance. Medical entomologists and vector control professionals will be able to refer to this review to advocate for tailored entomological surveillance strategies.

The main arboviruses covered by in this review are dengue virus (DENV), chikungunya virus (CHIKV), Zika virus (ZIKV), West Nile virus (WNV), and Rift Valley fever virus (RVFV). Whereas the first three are transmitted by recently established populations of invasive mosquitoes (*Aedes aegypti* and *Ae*. *albopictus*), WNV and RVFV are transmitted by native mosquito species.

We review here current knowledge generated by entomological surveillance of the main vectors of mosquito-borne arboviruses in the Mediterranean area and put forward proposals for harmonising the most appropriate surveillance activities for identified priorities.

## Methodology

**Fig 1:**
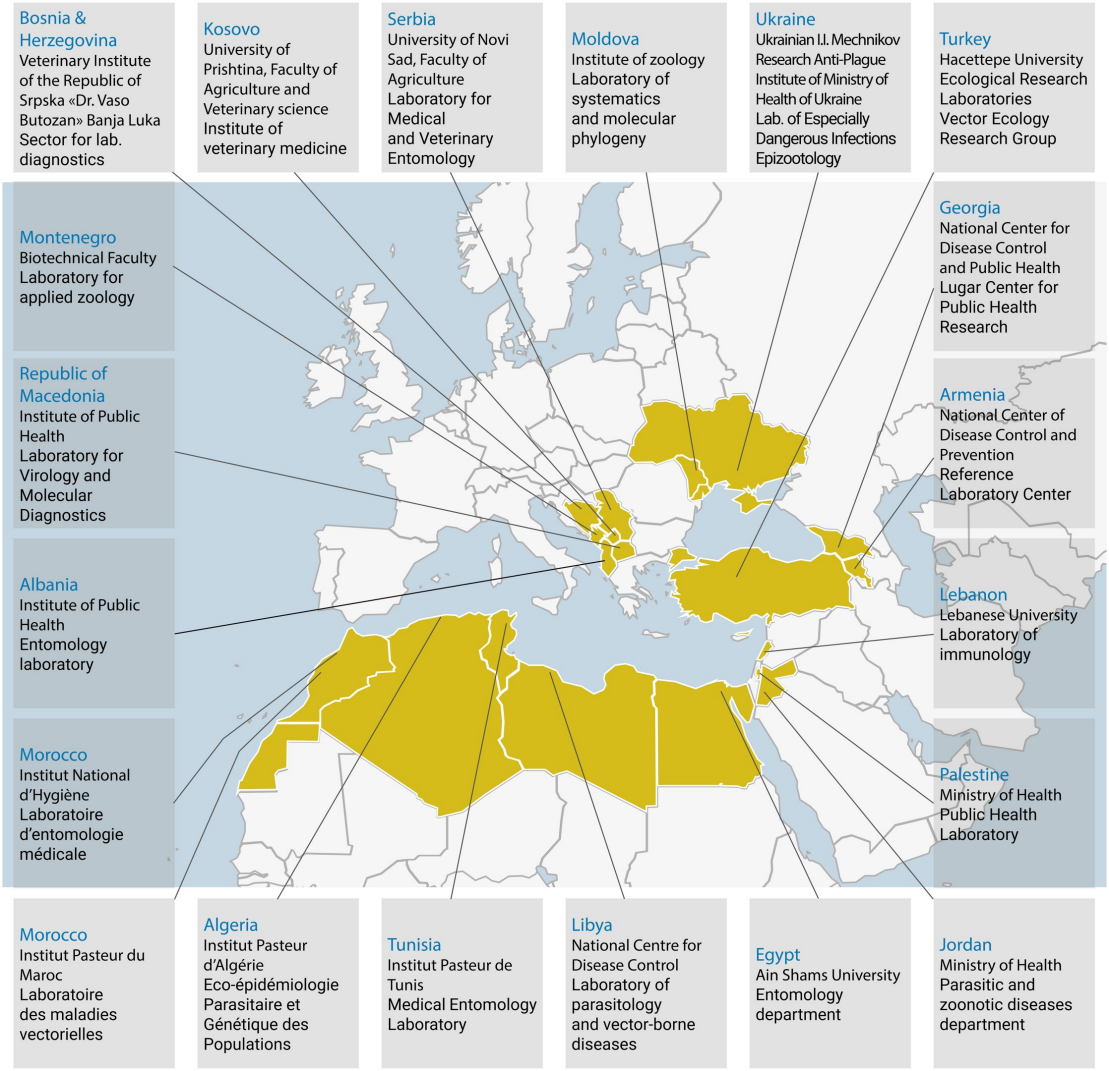
Map of the member countries of MediLabSecure and locations of the laboratories constituting the medical entomology network. The figure was originally published by Jourdain and colleagues [6]. Administrative boundaries: IRD, Cartographic service. IRD, French National Research Institute for Sustainable Development.

Literature-based knowledge, including grey literature from international bodies (European Centre for Disease Prevention and Control [ECDC], European Food Safety Authority [EFSA], World Health Organization [WHO]), on specific viruses (i.e., WNV, RVFV, DENV, CHIKV, and ZIKV) and invasive vectors was reviewed, with a focus on the Mediterranean area. In addition, experts from the 20 medical entomology laboratories of the MediLabSecure network were solicited. Nineteen laboratories responded and reviewed the national grey literature in their respective country (Fig 1 and S1 Table).

## Identified priorities for mosquito surveillance in the Mediterranean area

In the last 2 centuries, different mosquito-borne viruses have been reported in the Mediterranean Basin and the Black Sea area [4,7–10]. Global information on main vectors of arboviruses is presented in Table 1. For a precise understanding of the distribution of each species, the reader can refer to a recent publication focusing on Culicidae distribution in the Euro-Mediterranean area [11].

**Table 1:** Literature-based inventory of known and suspected arbovirus vectors in the Mediterranean area [4,7–11].

Family	Genus	Virus	Amplifying hosts	Geographic distribution	Known or suspected vectors in the area
Bunyaviridae	*Phlebovirus*	Rift Valley fever	Cattle, sheep, camels	Africa, Middle East	*Ae*. *aegypti* *Ae*. *albopictus* ***Ae*. *caspius*** *Ae*. *detritus* ***Ae*. *vexans (***°***)*** ***Cx*. *antennatus*** ***Cx*. *perexiguus*** ***Cx*. *pipiens s*.*l*.** *Cx*. *theileri* ***Cx*. *tritaeniorhynchus***
	*Orthobunyavirus*	Tahyna	Hares, rabbits, hedgehogs, rodents	Africa, Asia, Europe	***Ae*. *vexans*** *Ae*. *cinereus* *Ae*. *caspius* *Ae*. *cantans* *Ae*. *communis* *Ae*. *punctor* *Ae*. *flavescens* *Ae*. *excrucians* *Cs*. *annulata* *Cx*. *modestus* *Cx*. *pipiens s*.*l*. *An*. *hyrcanus s*.*l*.
Flaviviridae	*Flavivirus*	West Nile	Birds	Asia, Africa, Americas, Europe, Middle East, Oceania	*Ae*. *caspius* *Ae*. *vexans* *Ae*. *dorsalis* ***Cx*. *pipiens s*.*l*.** ***Cx*. *modestus*** *Cx*. *perexiguus* *Cx*. *theleiri* *Cx*. *tritaeniorhynchus* *Cx*. *univittatus* *Cq*. *richiardii*
		Usutu	Birds	Europe, Africa	*Ae*. *caspius* *Ae*. *detritus* *Ae*. *albopictus* *An*. *maculipennis s*.*l*. *Cx*. *perexiguus* ***Cx*. *pipiens s*.*l*.** *Cx*. *univittatus* *Cs*. *annulata*
		Dengue	Primates, **humans**	Cosmotropical	***Ae*. *aegypti*** ***Ae*. *albopictus***
		Zika	Primates, **humans**	Africa, Asia, Americas, Pacific	***Ae*. *aegypti*** ***Ae*. *albopictus***
		Yellow fever	Primates, **humans**	Africa, South America	***Ae*. *aegypti*** *Ae*. *albopictus*
Togaviridae	*Alphavirus*	Chikungunya	Primates, **humans**	Africa, Asia, Americas, Pacific	***Ae*. *aegypti*** ***Ae*. *albopictus***
		Sindbis	Birds	Asia, Africa, Australia, Europe, Middle East	***Ae*. *cinereus*** ***Ae*. *communis*** ***Ae*. *excrucians*** ***An*. *hyrcanus s*.*l*.** *Cx*. *theileri* *Cx*. *perexiguus* ***Cx*. *pipiens s*.*l*.** ***Cx*. *torrentium*** *Cx*. *tritaeniorhynchus* *Cx*. univittatus* Cs. *morsitans* ***Cq*. *richiardii***

Important vectors are indicated in bold.

(°) Transmission was reported for the subspecies *Ae*. *vexans arabiensis*.

*Considered a major vector of Sindbis virus in South Africa.

Abbreviations: *Ae*., *Aedes*; *An*., *Anopheles*; *Cq*., *Coquillettidia*; *Cs*., *Culiseta*; *Cx*., *Culex*; *s*.*l*., *sensu lato*.

The main invasive species in Europe have been identified in a previous review [12] and are relevant for the whole Mediterranean area [13]. *Ae*. *aegypti* and *Ae*. *albopictus* are considered the main invasive vectors because of their ability to transmit a wide variety of arboviruses, and they are subject to intensive surveillance efforts in the area. Distribution maps are updated several times a year by the European network of medical and veterinary entomologists (VectorNet), supported by ECDC and EFSA [14].

In accordance with the literature cited previously [1,3,4,7,9–14], reinforced by the opinion of our present group of authors, there is a consensus to address three priority entomological surveillance issues for the countries of the Mediterranean region: (1) invasive species as vectors of DENV, CHIKV, and ZIKV; (2) vectors of WNV; and (3) vectors of RVFV.

## General proposals for harmonising entomological surveillance

**Fig 2:**
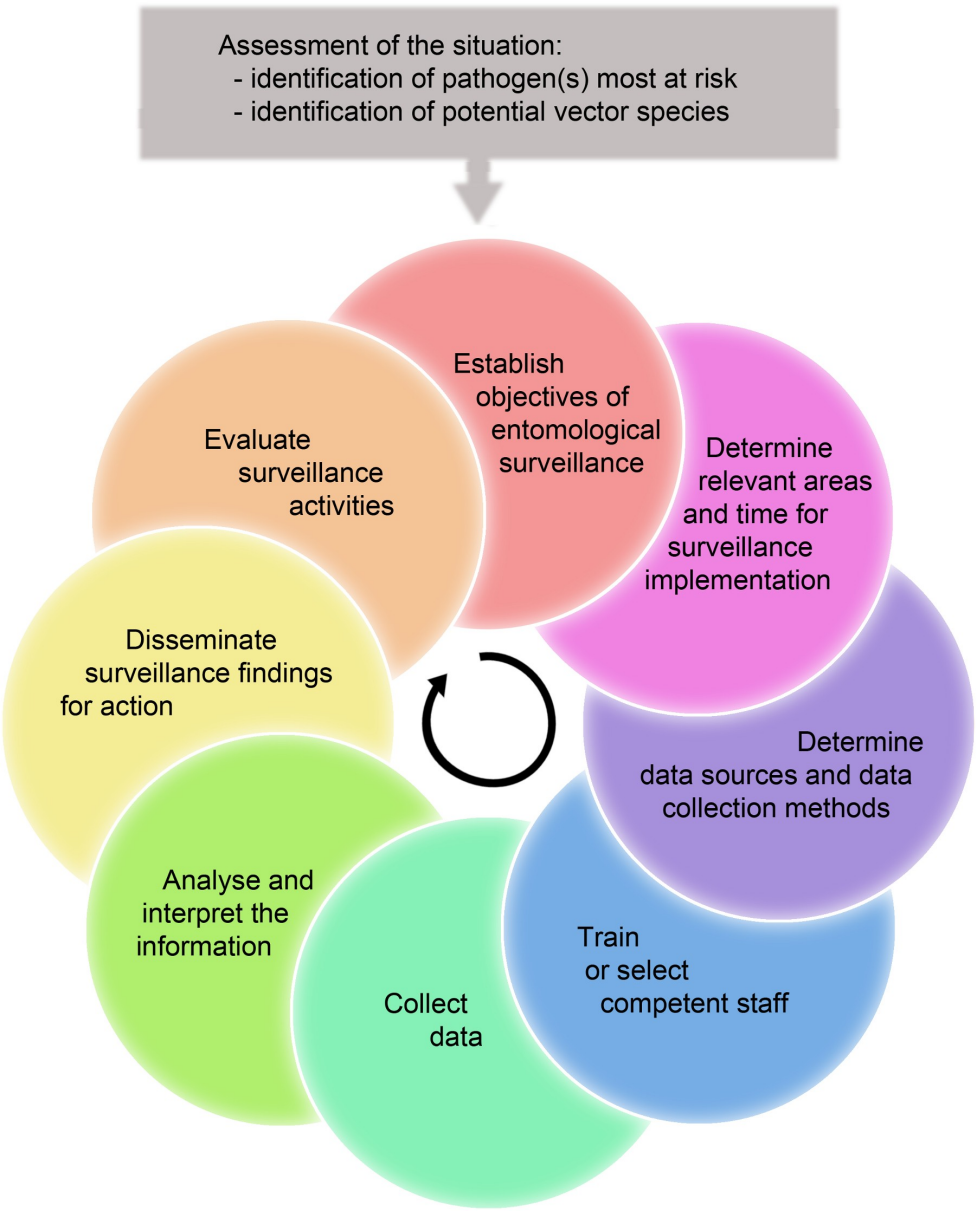
Process for implementing and updating entomological surveillance systems.

Entomological surveillance focusses specifically on collecting data on arthropods as vectors of human diseases and is subject to general principles of public health surveillance [15]. WHO defines it as a three-step process: (1) continuous, systematic collection of data; (2) analysis and interpretation of these data; and (3) dissemination of results to guide the planning, implementation, and evaluation of public health practises. As with every health system, entomological surveillance should be an adaptive process based on surveillance results and on evaluation of the system and potential changes in the entomological and epidemiological situation (Fig 2). Entomological surveillance activities (summarised in Box 1) have numerous, context-dependent objectives.

**Box 1. List of the main objectives of entomological surveillance of mosquito-borne viral diseases**

**The different objectives of entomological surveillance**

Entomological surveillance is defined and implemented to meet specific objectives; the main ones are as follows:

1. Risk assessment: Prioritisation of public health threats
  The purpose of risk assessment is to qualitatively or quantitatively determine the likelihood and impact of an identified threat (or hazard) to the environment, individuals, or populations. With infectious diseases, risk assessment is a step-by-step process that unfolds as follows: (1) identification of the hazard(s) posed by pathogens; (2) assessing the probability of pathogen introduction into a specific area by any possible route; (3) assessing the probability of transmission within the at-risk zone (assuming the presence of competent vectors, susceptible vertebrate hosts, and the biotic and abiotic conditions suitable for transmission); (4) assessing the probability of establishment, spread, and persistence of the disease; and (5) assessing the impacts on health and the economy. Risk assessment is by nature interdisciplinary and entomology provides key inputs for evaluating VBDs. De Vos and colleagues [16] have developed an interesting framework for assessing the risk of VBDs that can be easily transferred to human health.
2. Early warning systems
  Entomological surveillance can be used for early detection of viral circulation, before the emergence of the index case in a human and/or animal, and therefore paves the way for the preparation of response measures [17,18]. The critical features of early warning systems are sensitivity to detect outbreaks, specificity to avoid false negatives, and timely response to instigate early interventions [19]. The usefulness and relevance of mosquito-based arbovirus surveillance as a tool for developing early warning systems is situation dependent but seems particularly appropriate for endemic enzootic diseases, in which viral amplification in wild hosts and enzootic vectors precedes human cases. Surveillance should thus focus on areas known for sylvatic or synanthropic transmission cycles, which constitute hotspots for transmission, rather than cover a more extensive area [20]. Novel approaches have recently been developed, which mitigate some former limitations [21].
3. Identification of the vector species involved in a transmission event
  Two situations call for the implementation of entomological surveillance to identify the vector species involved in a specific transmission event. The first is when several potential vectors are present and established, and the role each species plays in transmission needs to be clarified in order to adapt control strategies. For example, where both *Ae*. *albopictus* and *Ae*. *aegypti* are present in an area, the pathogen could be transmitted by one or both of them and may depend on its viral genotype [22,23]. The second is the case of viral emergence in a new territory (e.g., ZIKV in the Americas), where it is essential to identify the vector(s) involved in order to optimise the response strategy. The possibility of the virus being transmitted by unknown vectors for a particular pathogen should not be overlooked [24].
4. Identification of the circulating pathogen strain
  In some cases, viral detection in the vectors is an effective way to identify the circulating virus or strain. This can be particularly useful for viruses that are very difficult to detect in the blood of their vertebrate hosts because of very low viremia levels (e.g., WNV). This will contribute to a better understanding of the vector–host–pathogen system, in particular virus–vector interactions (vector competence, duration of the extrinsic incubation period, etc.). Detection of WNV lineage 2 for the first time in pools of *Culex pipiens* in northern Italy [25] shows the contribution entomological surveillance can make here.
  This objective is important for viruses such as DENV or CHIKV, as it is generally easier to obtain these viral materials in humans.
5. Optimising vector control in time and space
  Knowledge of the spatial and temporal dynamics of mosquito populations provides crucial information for characterising areas and seasons that may be most at risk of disease transmission [26–28].
  The population dynamics of certain mosquitoes can vary considerably from region to region and year to year under pressure from different factors, including climate, land cover, agricultural practises, and water management. Urban species exhibit strong density heterogeneity across districts of the same town/city.
6. Guidance for source reduction campaigns
  In addition to the points mentioned in the previous paragraph, characterising breeding sites will improve source reduction campaigns, whether these concern larval control undertaken by mosquito control operators or by the social mobilisation actions of communities, especially in domestic and peri-domestic breeding sites (which are most often of anthropogenic origin). In this context, a typology of aquatic sites can be constructed to focus attention on the most common and productive sites [29]. Local specificity and private and public lands must be taken into account (e.g., [30]).
  Larval control through environmental management, larviciding, biological control, and social mobilisation are key components of a proactive and sustainable vector control strategy and must be pursued where a health threat event occurs. In the case of viruses transmitted by *Stegomyia*, coverage of the intervention is crucial, and source reduction campaigns should rely heavily on community mobilisation [31,32]. However, examples of social mobilisation actions from the Mediterranean area and, more broadly, outside of tropical countries remain rare [33,34] and reflect the need to promote horizontal approaches and no longer rely only on top-down strategies.
7. Insecticide resistance monitoring
  Most vector control programmes rely to a large extent on chemical insecticides, so monitoring vector susceptibility to commonly used active substances should be a key component of entomological surveillance and an integral part of these programmes [35]. Knowledge of the status, changing trends, and distribution of resistance in vectors is a basic prerequisite to guide policy and operational decisions, which involve choosing appropriate insecticides and implementing comprehensive resistance management strategies. Decision-making needs to be based on reliable vector susceptibility data, which call for standardised monitoring. Information on insecticide resistance is highly important in order to identify areas where resistance may jeopardise VBD management [36].
8. Evaluating the efficacy of vector control
  Health authorities and local medical entomology laboratories can carry out entomological surveillance in order to evaluate the efficacy of control measures. In this case, monitoring will focus on the species and developmental stages targeted by the control actions. However, evaluations of control programmes will differ depending on whether the focus is source reduction or adult control. Source reduction is a preventive measure aimed at reducing larval populations through biological or chemical control or by eliminating mosquito breeding sites. Such actions are planned in advance so that proper evaluation measures based on larval and/or pupal indices can be put in place [37]. Adult mosquito monitoring is more challenging, as sampling results are less reproducible and actions are most often carried out in reaction to an unexpected event. It is therefore more difficult to conduct proper evaluation studies on a routine basis. In this case, monitoring will mainly be considered operational research dedicated to assessing the effectiveness of the vector control method and will require standardised, high-quality studies optimally based on entomological and epidemiological metrics [31].

### General framework: Towards global, integrated surveillance

Implementation of entomological surveillance systems is impeded by several factors, such as limited investment, lack of human resources, limited entomology capacities, difficulties in standardising data collection, and concerns for the economic impact on the tourism sector [38]. These challenges may be partly addressed by implementing a global, integrated surveillance framework in the prism of the One Health strategy in order to better structure management strategies and coordination, to promote intersectoral approaches, and to stimulate the sharing of financial and human resources.

This approach requires surveillance systems in which the sectors concerned (entomology, human and animal health) interact and work together to improve public health at all levels: locally, nationally, and globally. It is essential to broaden expertise and skills to include the ecological and environmental sciences and to incorporate the different disciplines involved in implementing health policy.

This interdisciplinary approach should go beyond surveillance activities to consider the impact of control strategies on vertebrate hosts of VBDs and also the consequences for the environment and ecosystems. The development of multidisciplinary networks will provide the means to address these challenges and, more specifically, to promote integrated surveillance strategies and effective sharing of knowledge and information [5].

The involvement of numerous disciplines calls for a formal coordination framework closely monitored by a steering committee. A common framework does not imply uniformity, as there is no one-size-fits-all surveillance system. Surveillance strategies should be designed for specific entomological and epidemiological situations. Situation analysis and the proposed integrative approach can help define priorities and surveillance objectives.

The rational process for surveillance should be based on the key elements of any integrated vector management (IVM) strategy: advocacy, capacity building, collaboration within the health sector and with other sectors, and evidence-based decision-making [39].

Dente and colleagues’ [40] conceptual framework stipulates the criteria for assessing the integration of systems for the surveillance of arboviral diseases at the following levels: policy and institutional, data collection and analysis, and dissemination of results. This framework could be useful not only for evaluating surveillance systems but also to improve preparedness and response to arboviruses of concern.

The situation regarding mosquito-borne viruses has evolved over recent decades. For example, novel lineages of WNV have emerged and modified the global epidemiology of the disease, whereas RVFV has expanded geographically. Other diseases, such as dengue or chikungunya, are no longer geographically restricted to the tropics and have a strong urban component as a result of urbanisation, global trade, and travel. Viruses such as DENV, CHIKV, and ZIKV have spread worldwide to an unprecedented degree [1]. Other candidates for emergence have been identified, such as Mayaro and Oropouche viruses [41]. These general observations call for the reinforcement and coordination of surveillance on a global scale, enabling the exchange, integration, and use of surveillance data [38]. This means standardising the collection of data and recording and storing it in a format that is easy to access, use, and share [36,42].

Although control measures are not part of the surveillance process, both are closely linked, as epidemiological and entomological surveillance activities are the backbone of effective control campaigns. There is, therefore, a pressing need to develop response capacities, in particular vector control, in parallel with the optimisation of surveillance systems, according to the IVM framework promoted by WHO [13,39]. This type of investment is also a precondition for the sustainability of surveillance systems.

### Entomological and epidemiological scenarios

Like every public health action, entomological surveillance must be tailored to the entomological and epidemiological situation (presence of efficient vector population[s], presence of susceptible host[s], and pathogen introduction or intensity of circulation) [43].

The risk of a given VBD can move from an initial entomological risk (presence of a competent vector) to a subsequent epidemiological risk (risk of transmission) when a vector is present. In order to differentiate entomological from epidemiological risk, different scenarios have been proposed depending on whether the mosquito species concerned is invasive or native [10,12,13]. Tailored and graduated actions of vector surveillance and control arise from these different scenarios (Table 2).

**Table 2:** Objectives of surveillance and possible public health actions based on entomological and epidemiological scenarios. Adapted from [10,12].

Scenario	Purpose of surveillance	Actions based on surveillance results	Supporting references
A1 Prior to introduction or establishment of invasive species	Surveillance of main routes of introduction: -Points of entry -Major communication routes linked to areas known to be or suspected of being colonised -At-risk activities -Main tourist areas	Vector control for local elimination of invasive species, especially in the case of newly introduced populations Raise public awareness to report the presence of invasive species and adapt behaviour	[44,45]
A2 The invasive species is locally established	Surveillance of the spread	Implement epidemiological surveillance in the colonised area	[46–48]
	Surveillance of seasonal dynamics	Identify seasons with possible risk Adjust planning in time	[27,47,49]
	Typology and productivity of breeding sites and/or breeding ecology	Guide larval control, including social mobilisation and targeting of the most productive breeding sites, door-to-door surveys Evaluate the efficacy of source reduction and larval control	[30,33,49]
	Define entomological parameters for estimating vectorial capacity	Update risk assessment	[16,50]
	Surveillance for abundances in colonised areas and identification of hotspots (i.e., areas with high adult mosquito abundances)	Prioritise vector control	[51,52]
	Surveillance of insecticide resistance	Guide vector control by choosing appropriate insecticides	[53–55]
B1 Vector is widely established: Emerging situations	Surveillance of seasonal dynamics: adult sampling to estimate adult mosquito abundances	Identify at-risk seasons and periods Evaluate effectiveness of insecticide treatments	[45,47,51,56]
	Larval surveys to identify key breeding sites	Guide vector control	[30,33,49]
B2 Vector is widely established: Endemic situations	Pathogen screening Entomological surveys to identify key breeding sites and hotspots of adult mosquitoes	Early warning system for emerging events, Identify serotypes or lineage Guide vector control and prioritise intervention areas	[18,42,57–59]
B3 Vector is widely established: Epidemic situations	Entomological investigation around cases (can be performed at the same time as active research of cases/door-to-door surveys)	Vector control Guide mechanical control of breeding sites on public lands Evaluate effectiveness of insecticide treatments	[60–62]

### Basic knowledge of the entomological fauna

Basic knowledge of the mosquito fauna and its spatial and temporal heterogeneity is required to assess risk and to implement targeted surveillance or vector control actions that are tailored and proportionate to the risks.

First, an inventory of the species present in the area in question is needed to identify the species that are known to be putative vectors. A distinction should be made between potential and proven vectors [63]. Using captures in different areas and during different periods of transmission, it is possible to draw up a specific inventory of the species in at-risk areas in terms of presence/absence as well as abundance in function of time and/or spatial units. In addition to this inventory of native and established species, the invasive species most likely to be introduced should be identified.

Once the main vector candidates are identified, the next step is to obtain reliable information on the various entomological population variables impacting virus transmission. Some of these data can be retrieved from the literature, but in most cases, risk estimation is much more precise when based on studies of local vector populations. The most important variables to be considered are blood-feeding behaviour, longevity, extrinsic incubation period for relevant pathogens, abundance, dispersal, etc. [50]. Knowledge of the seasonal dynamics and spatial distribution of vectors is useful for risk assessment and targeted risk management activities over time and space.

As most vector control programmes rely to a large extent on chemical insecticides, the monitoring of vector susceptibility to commonly used active substances should be a key component of entomological surveillance and an integral part of these programmes [35]. Knowledge of the status, changing trends, and distribution of insecticide resistance in vectors is a basic prerequisite to guiding policy and operational decisions, as it underlies the choice of appropriate insecticides and implementation of comprehensive resistance management strategies [36].

Following these considerations, which are generally applied, specific surveillance activities will be implemented according to the pursued objectives (Box 1) and the targeted vectors. The general framework is subsequently declined for three major issues in the region—i.e., invasive species as vectors of DENV, CHIKV, and ZIKV; vectors of WNV; and vectors of RVFV.

## Dengue, chikungunya, and Zika: Surveillance of invasive species

### Context

DENV, CHIKV, and ZIKV are transmitted mainly in urban settings by mosquitoes of the *Stegomyia* subgenus. The most efficient vectors are *Ae*. *aegypti* and, to a lesser extent, *Ae*. *albopictus*. *Ae*. *cretinus* is the only other *Stegomyia* present in the Mediterranean region [64], but as little information is available on its capacity to transmit viruses, this species will not be considered in this review. Another *Aedes*-borne virus, yellow fever virus (YFV), has also reemerged in Africa and South America over the last 4 decades. If YFV is introduced, *Ae*. *aegypti* and *Ae*. *albopictus* may be able to maintain an autochthonous transmission cycle, so surveillance of these invasive mosquito species will help improve preparedness and response to these different agents of virus transmission.

The design of the surveillance system will depend on the level of colonisation by one or several invasive species within the country and in neighbouring countries, as well as on the objectives of the surveillance (see Table 2 and Box 1).

### Surveillance of the introduction and establishment of *Ae*. *albopictus* and *Ae*. *aegypti*

The main objective of entomological surveillance in this context is to detect the introduction of invasive species, so the main focus will be the potential routes of introduction and dispersal. In the specific case of invasive *Aedes* species, there are two main modes of dispersal. On an intercontinental scale, dispersion mainly occurs at the egg stage and is associated with the international trade in specific goods likely to introduce a sufficient number of individuals that might survive and subsequently reproduce, mainly used tyres and, to a lesser extent, lucky bamboo [12]. On an intracontinental scale, the species gradually spreads along major communication routes in association with traffic, especially when the mosquitoes have a natural ability to enter vehicles, which is common for *Ae*. *albopictus* [65].

*Ae*. *aegypti* may have different introduction pathways, as the species was broadly spread over the Mediterranean Basin until the 1950s [64]. Recent genetic evidence suggests that remnant populations persist around the Black Sea region [66], with a recent spread into northeastern Turkey [67].

Given the key routes of invasion, a typology of high-risk sites for invasive species introduction can be drawn up based on presence–absence models or by modelling environmental and demographic variables. These sites, which include points of entry (ports, airports, and ground crossings), parking areas and resting places along main communication axes, rail freight interchanges, railway nodes, used tyre storage sites, greenhouses containing imported exotic plants, and green urban spaces, should be selected for targeted surveillance.

Trapping locations can be prioritised on the basis of data on traffic, the volume of imported goods, and the vicinity of the colonised area [12,45]. Ovitraps are the main tool used for the surveillance of invasive mosquito introduction. ECDC guidelines [12] contain useful technical information about trapping procedures and modalities (type, frequency, density, period of trapping). Ovitraps are less sensitive at certain locations, such as used tyre storage sites or greenhouses, because their large numbers of available oviposition sites compete with the traps. Entomological surveys should preferably be carried out on a regular basis (2 to 4 per year) at these locations.

Modelling using mainly abiotic (climate, photoperiod) and landscape (urbanisation) variables can help identify the most suitable areas for invasive species establishment. Different methods have been used to conduct this type of risk assessment for *Ae*. *albopictus* and *Ae*. *aegypti* (e.g., [68–70]).

*Ae*. *albopictus* is likely to be introduced and become established in urbanised areas owing to its affinity with the human environment. A plethora of artificial breeding sites compete with ovitraps in this type of environment, so sensitivity is again an issue. Early detection of introduction in at-risk areas, such as urban or peri-urban locations, is therefore a challenge. Passive surveillance, in which the general public are involved in detecting and reporting invasive species, is a particularly promising and economically advantageous way to address the problem of ‘random’ introduction in urbanised areas or at a considerable distance from the colonised area [71,72]. In most cases, identification of *Stegomyia* is feasible from a mere photo, whereas the main challenges relate to the organisation and formalisation of a reporting system and to making it widely known and accessible, especially to other countries. For example, a unique platform (web portal) could return reports to the competent regional authority and would also enable the development of a regional dynamic through cooperation and data sharing, a crucial aspect of the process of surveillance of invasive species.

### Surveillance in areas with locally established populations of *Ae*. *albopictus* or *Ae*. *aegypti*

Once an invasive species is established, entomological surveillance can assume different objectives.

#### Monitoring the spread

Precise knowledge of the vector distribution is needed to support the implementation of epidemiological surveillance and the decision to trigger vector control programmes. The aim of monitoring species distribution in areas with locally established populations is to rationalise vector control around imported arboviral cases, brought in mostly by viremic travellers coming back from endemic or epidemic countries. Surveillance should, therefore, be concentrated in the most densely populated areas and in major tourist locations, where imported cases are most likely to occur.

The system for monitoring spread will be similar to that for the surveillance of introduction. It will mainly consist in deploying a network of ovitraps according to the previously mentioned criteria in proximity to the colonised area.

The use of passive surveillance will be crucial in this context, given the previously highlighted difficulties.

There is usually a latitudinal and altitudinal limit above which a species is unable to establish a population. For example, an average January temperature of 0 °C is usually considered the survival threshold for *Ae*. *albopictus* diapausing eggs [73], although this figure should be treated with caution in urban areas, as they contain many microclimates with higher temperatures. For example, the species was present in Trento, Italy, despite minimum temperatures as low as −10 °C and an average January temperature of −5 °C being recorded [74]. One way of estimating altitudinal limits is by monitoring the establishment of species along altitudinal transects.

#### Estimating vector density

Estimating vector density is a major element in providing guidance for vector control, but it is difficult to do on a routine basis. Sampling immature stages (larval indices, such as Breteau, container and house, or pupae-per-person and pupae-per-hectare indices) is still widely used to estimate vector density [75]. These container-based indices exhibit weak correlations with the density of biting mosquitoes per human and are poor indicators of the risk of arbovirus transmission [76]. The value of ovitraps for this purpose is under discussion and undoubtedly depends on the context, especially where there are numerous competing breeding sites, given the skip-oviposition behaviour of *Stegomyia* [77].

For these reasons, adult sampling remains the method of choice to estimate adult density. Of the various traps available, the BG-Sentinel trap with lures (BG trap; Biogents, Regensburg, Germany) is currently the gold standard for monitoring adult populations of *Aedes* spp. [78,79]. However, BG traps are costly, and collection is labour intensive, making it unrealistic to deploy them on a large scale. Gravid *Aedes* traps (GATs) may be an alternative, but more studies are needed to confirm their effectiveness for routine surveillance purposes [80].

A pragmatic approach to estimating adult density would be to develop fine-scale models based on land cover, land use, meteorological, and sociodemographic data (review in [81]) in order to target interventions in locations with high densities of mosquito populations [51].

#### Seasonal dynamics

Thorough knowledge of seasonal dynamics is useful for risk assessment and risk management purposes, and in particular, for determining the human surveillance period and identifying the months with the highest entomological risks.

The seasonal dynamic in a particular region can be assessed using a dense network of ovitraps or BG traps [12,27].

This dynamic may, however, vary according to the climate. The photoperiod and, hence, the latitude are key factors in *Ae*. *albopictus* diapause. Within a given country, it may be appropriate to record these data in all climatic regions.

#### Guidance for vector control actions

Once an invasive species is established, only routine larval control strategies are feasible, as elimination is almost unattainable, at least in continental areas and with the vector control tools that are currently available [64].

Entomological surveillance will help in assessing larval source reduction strategies and social mobilisation actions. Larval surveys may also be of interest and can be implemented simultaneously. A typology of breeding sites could be drawn up from larval surveys carried out on private and public lands [30]. Larval and pupal surveys can be used to identify the most commonly encountered and most productive breeding sites, which will then become the focus of public actions and key messages mobilising the community in order to achieve the greatest impact on the adult mosquito population [75]. Larval surveys should, as far as possible, take into account the contribution of cryptic larval breeding sites [82]. This kind of investigation can then contribute to identifying local specificities concerning aquatic sites and hence to implementing targeted actions [83–85].

Surveillance efforts will focus on areas and locations that present the greatest risk to public health (history of high densities, public nuisance complaints, hospitals, automotive recycling yards, tyre storage, neighbourhoods with green areas favouring shade and with the presence of containers, or significant presence of water storage).

Once established, it will be necessary to evaluate the susceptibility of the mosquito population to commonly used insecticides according to standardised guidelines [35].

### Surveillance of widely established populations of *Ae*. *albopictus* or *Ae*. *aegypti*

When populations of invasive mosquitoes are widely established, most of the activities described in the previous scenario (local establishment) will continue. The main supplementary activities concern guidance and evaluation of the vector control activities implemented around imported arboviral cases [86].

The effectiveness of vector control measures can be assessed by trapping adults in the intervention area before and after these measures are carried out. Ideally, trapping should also be carried out according to a randomised design in nontreated areas presenting similar environments to the treated areas [31].

### Evidence of autochthonous virus transmission

The general objectives of entomological surveillance here are substantially the same as in the previous scenario, but the modalities will have to be adapted to vector control actions, as in the event of viral circulation, these will target adult populations in addition to source reduction.

In the case of autochthonous transmission, mosquitoes can be captured for virus screening. This is particularly important when both *Ae*. *albopictus* and *Ae*. *aegypti* are present in order to identify the main vector(s) involved in the transmission event and to adapt the strategy of vector control. Because of low virus prevalence in mosquitoes, a large number of mosquitoes, promptly collected in the direct vicinity of arboviral cases, need to be screened [87]. Mosquito-based surveillance is unlikely to make a useful contribution to early warning systems for *Stegomyia-*borne virus introduction given the previous consideration and the random nature of the geographical distribution of imported cases. In this case, it is more efficient to rely on human surveillance.

## West Nile fever

### Context

**Fig 3:**
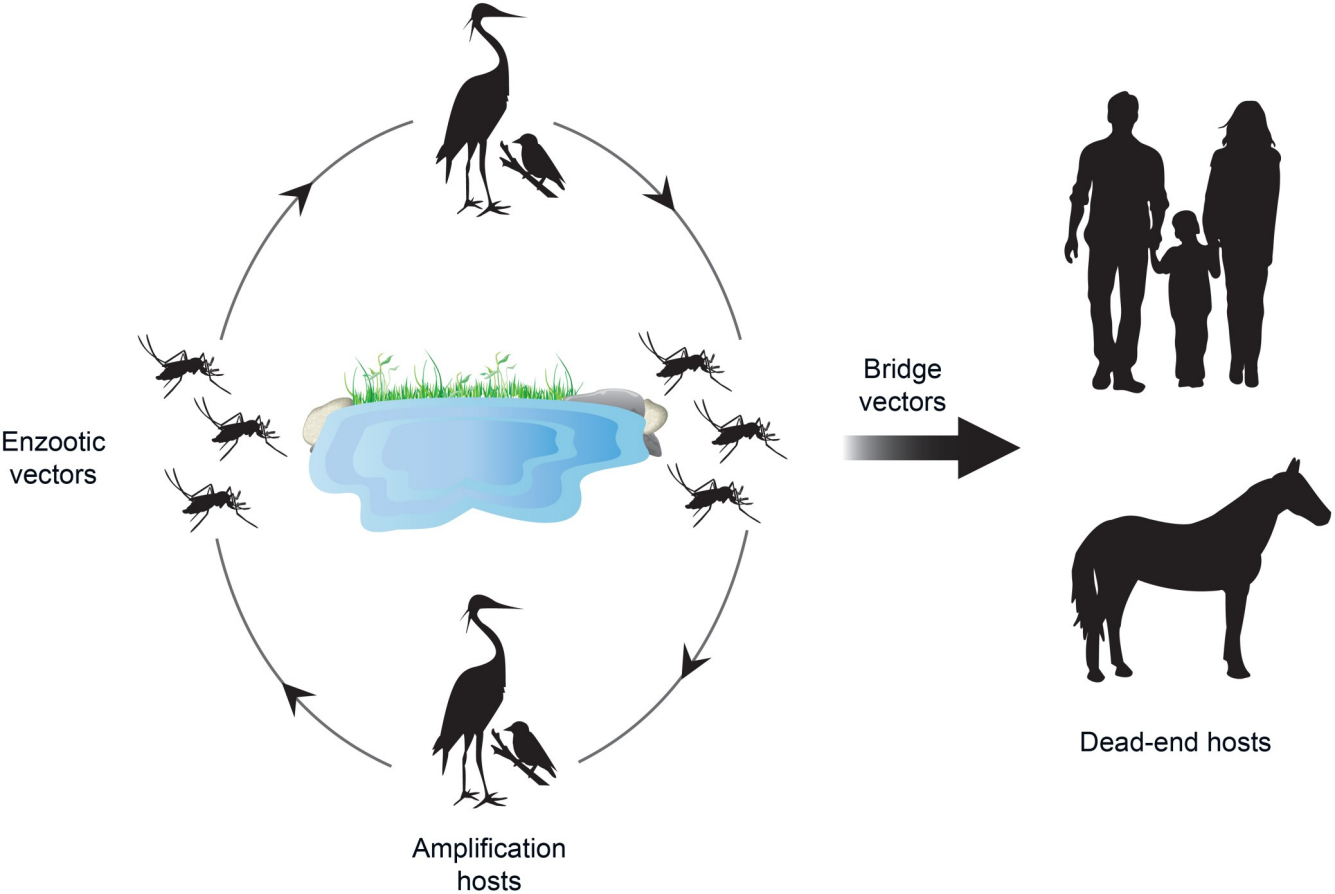
Epidemiological cycle of West Nile virus.

WNV is one of the most widely distributed flaviviruses and the main cause of arboviral human encephalitis worldwide [88]. It is a zoonotic arbovirus maintained in nature in an enzootic cycle that involves birds, mostly passerines, and ornithophilic mosquitoes of the genus *Culex* (Fig 3). Birds serve as amplifying hosts of the virus and contribute to its dispersal. The disease can also affect humans and horses, but they are considered dead-end hosts, meaning that although they become infected, the level of viremia is insufficient to infect mosquitoes and hence spread the disease.

WNV lineages are genetically and geographically diverse [89], and extensive data on WNV circulation in the Mediterranean are available (e.g., [4,90–93]). Historically, lineage 1 (clade 1a) was the most widespread lineage around the Mediterranean Basin. However, the disease pattern has changed over the last 2 decades with the emergence in 2004 of lineage 2 in eastern Europe (Hungary), which has since become endemic throughout eastern, central, and southern Europe. Lineage 2 strains have caused the majority of human and equine neurological cases recently reported in Europe, as well as unexpected avian mortality. Other lineages have been isolated from mosquitoes collected in the Mediterranean area (lineage 3 in the Czech Republic, lineage 4 in southern Spain and Austria), but they have not, so far, been associated with human or animal diseases [89,91,94]. Cocirculation of different lineages across Mediterranean countries provides an opportunity for genetic assortment and the emergence of new strains [95].

### Risk-based approach

Many factors have been suggested to explain WNV spread. Temperature is one of the most important drivers in WNV transmission, and above-average summer temperatures have been associated with human and equine cases in Europe [96,97]. Regarding early detection, wetlands with wide avian diversity located along the major flyways of migratory birds are potential hotspots for monitoring enzootic activity [98]. Other environmental risk factors have been assessed, including the normalised difference vegetation index (NDVI), avian biodiversity, land use, and landscape composition, and these have been reviewed for the Mediterranean Basin [99]. Predictive models for the Mediterranean area [96,97,100], Morocco [101], and Tunisia [102,103] have also been built.

### Surveillance

WNV surveillance requires an integrated approach that takes into account the enzootic, epizootic, and epidemic transmission cycles of the virus. However, the proportionate design of a surveillance system is country dependent and needs to consider the environmental conditions and local epidemiology of the disease [104]. Several Mediterranean countries have implemented integrated WNV surveillance systems [105,106]. The entomological component of most European surveillance systems has been reviewed by Engler and colleagues [42].

Given that human and equine cases appear to be the result of a spillover from the enzootic cycle, vector surveillance can serve as an early warning system, anticipating viral circulation several weeks before the onset of symptoms in humans, as demonstrated in Greece, Italy, and Serbia [18,107–110].

Entomological surveillance can enhance the sensitivity, early detection capability, and spatial specificity of WNV surveillance systems [18] and provide support in implementing risk management measures, such as raising awareness among clinicians to improve diagnosis, educating the public in appropriate personal protection against mosquito bites, and screening blood to reduce virus transmission by transfusion [111]. Entomological surveillance together with ornithological surveillance has been assessed as a more cost-effective strategy for blood and organ safety compared with systematic screening in regions where infected humans or equids were detected in the previous year, as demonstrated in Italy [18].

A further advantage of mosquito surveillance is the possibility to identify new viral strains/lineages or other arboviruses—such as Usutu virus (USUV)—at little additional cost [18,112,113].

There are some limitations to mosquito-based WNV surveillance for early detection [105,109,114]. It is costly and labour intensive, and significant sampling and testing efforts are required, as the number of mosquito pools submitted for virus detection appears to be critical for the sensitivity of this surveillance system. Moreover, results have to be delivered quickly so that vector control actions can be promptly implemented. Because of these constraints, mosquito-based early warning surveillance has been considered of little value in countries where the virus has been circulating at a low level, such as France, Spain, and Israel [105].

To overcome these disadvantages, entomological surveillance must include evaluation of the environmental and epidemiological context. It should be (1) based on knowledge of vector population seasonal dynamics, (2) implemented/intensified when risk is increasing (greater virus circulation compared with the previous year or neighbouring countries/areas), and (3) focussed on high-risk areas identified by biotic and abiotic factors, as mentioned previously.

Entomological surveillance can, however, have other objectives. Monitoring mosquito populations advances knowledge of disease cycles [8] and significantly improves the spatiotemporal risk assessment process and preparedness [42].

Mosquitoes can be collected in areas either at risk of or of known viral circulation to improve knowledge of the species involved in transmission and identify their breeding ecology, abundances, distributions, and trophic preferences [8,115,116].

Adult stages are most often targeted, but larval sampling can usefully complement entomological monitoring [42]. Most often, sampling is carried out monthly during the period of vector activity. However, weekly to biweekly adult sampling seems to be more appropriate if the objective is a sensitive early warning system [18,108,109].

## Rift Valley fever

### Context

**Fig 4:**
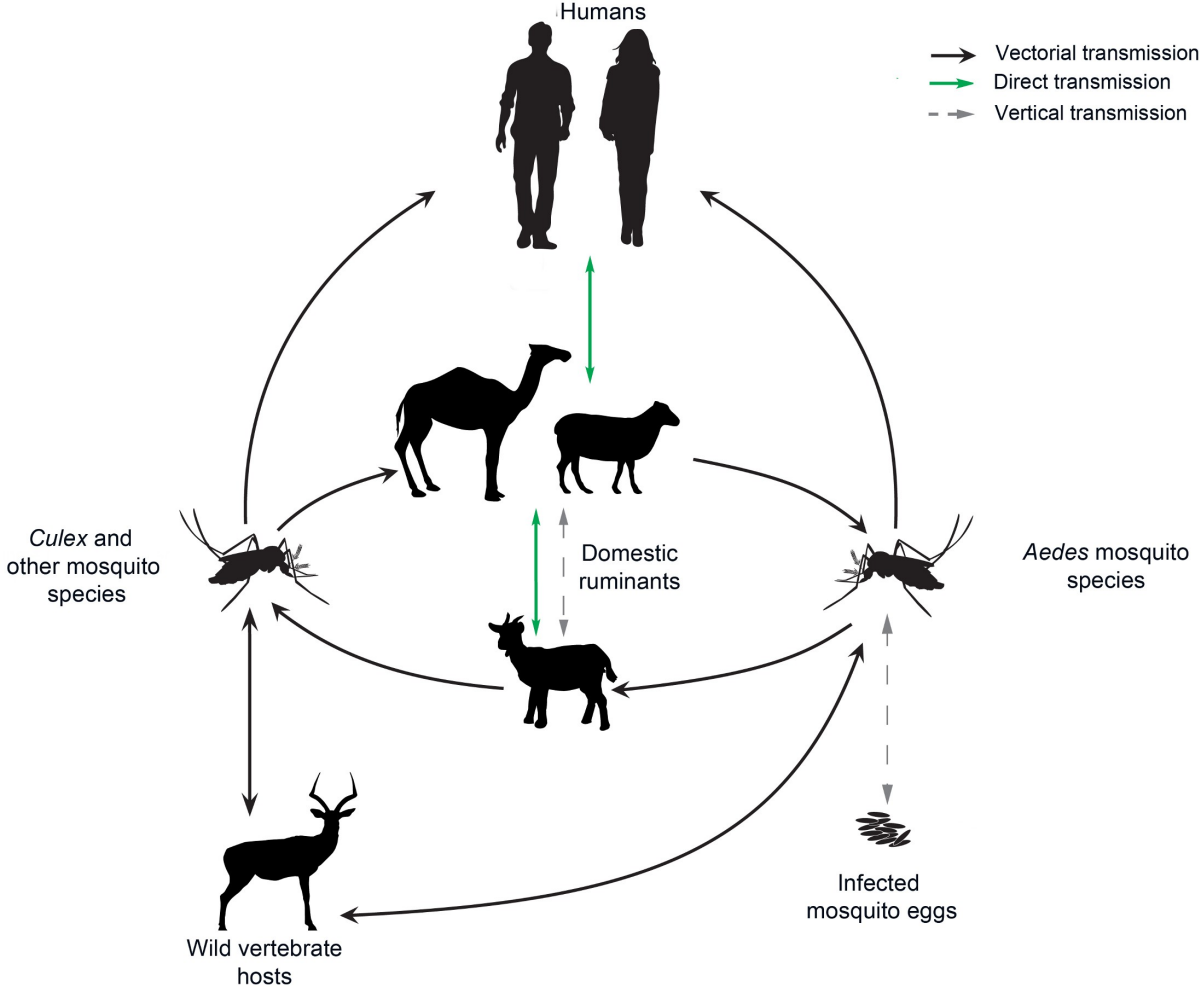
Epidemiological cycle of Rift Valley fever virus. *The figure is adapted from [119]*.

RVFV emergence may have a considerable impact on both humans and livestock. It is a complex VBD, as several vertebrate hosts can be affected, different transmission routes are possible, and there are also multiple pathways of virus introduction that are highly dependent on the local context (Fig 4). Further complexity lies in the fact that RVFV is readily transmitted through a broad range of mosquito genera and by other vectors, including sand flies and ticks [117]. Mosquito species of the *Culex* and *Aedes* genus are considered the most competent vectors. The following species are associated with RVFV transmission in the Mediterranean area: *Ae*. *vexans*, *Ae*. *caspius*, *Ae*. *detritus*, *Culex pipiens*, *Cx*. *theileri*, *Cx*. *perexiguus*, *Cx*. *antennatus*, *Cx*. *tritaeniorhynchus*, and *Ae*. *albopictus* [9]. The presence of *Ae*. *aegypti* in Egypt and around the Black Sea in Russia, Georgia, and Turkey [67,118] justifies its inclusion in the list.

Historically, RVFV was geographically limited to sub-Saharan eastern Africa, especially the Rift Valley of Kenya and Tanzania. In recent decades, the geographical range of the disease has expanded [3,9,120], and regular incursions of the virus have been observed along the Nile River in Egypt. Serological investigations in humans and animals indicate viral circulation apart from epidemic events [121]. Other serological studies suggest RVFV is circulating in areas of Algeria, Morocco, Tunisia, and Libya [4,122]. The probability of RVFV being introduced into Europe has been assessed as very low [123].

Livestock movements are the main factor for introducing the virus into disease-free areas. Camels crossing the border from Sudan to Egypt and the sheep trade between sub-Saharan Africa and northern Africa have been implicated [124]. The spread of the disease to Europe is, however, controlled, as there is a ban on livestock trade from the Middle East and northern Africa, although illegal importations cannot be excluded, particularly in central and southern Europe. These factors justify implementing dedicated systems of surveillance of potential RVFV vectors, acquiring knowledge of their bionomics, and carrying out vector competence studies [123].

### Risk-based approach

In the interests of efficiency and efficacy, risk-based surveillance should focus on potential hotspots and periods of disease introduction and transmission [125]. The EFSA recommend developing early warning systems based on epidemic intelligence and predictive models [123]. Models based on rainfall, sea surface temperature, and NDVI have been developed, although their area of validity is restricted to East Africa. Geographically extending these models to other areas (including the Mediterranean Basin) is problematic because of differences in climate and environmental drivers and the epidemiology of the disease [124]. Areas of the Mediterranean Basin at higher risk for RVFV can be identified through analysis of animal trade data and the distributions of competent vectors and susceptible hosts.

Distribution models of RVFV vectors around the Mediterranean Basin have been developed [9], and risk maps for identifying suitable areas for Rift Valley fever emergence are also available for the Maghreb region [126], Egypt [127], Spain [128], and Italy [129]. Potential hotspots have been identified in northern Africa, from Morocco to Libya [126], and include farming areas and desert oases, especially those located close to water bodies and animal facilities. In Egypt, the risk of virus introduction stems from camel importation from Sudan, whereas the risk of transmission is heightened in animal markets and slaughterhouses, most commonly located in crowded areas [127].

### Surveillance

RVFV is a perfect example of an arbovirus requiring an integrated approach consistent with the One Health framework to make best use of scientific capacities and resources [130].

Emphasis should be placed on animal and human surveillance, including passive surveillance of animal abortions, sentinel herds around the Mediterranean Basin, and during high-risk periods, as well as human laboratory-based surveillance [123,126]. In addition, entomological surveillance efforts should be focussed on the areas at highest risk of virus introduction and transmission. In spatial terms, entomological surveillance should preferably be carried out in the vicinity of the previously mentioned areas at higher risk of RVFV transmission (quarantines, slaughterhouses, and animal markets). The illegal movement of animals calls for surveillance to be strengthened in animal facilities along borders with endemic countries. In terms of time, entomological surveillance should be reinforced in specific countries in three cases: (1) during viral circulation in neighbouring countries, (2) during the weeks preceding the Islamic festival Eid al-Adha, when there is increase in animal trade, and (3) during periods of heavy rains (especially in natural biotopes).

Targeted entomological surveillance will guide vector control actions, such as source reduction and larviciding, and will raise the awareness of residents and professionals in the livestock sector in at-risk areas.

Regarding entomological scientific issues, the seasonal dynamics of the main vectors should be assessed in different areas vulnerable to virus introduction and transmission. Additional studies can easily be devoted to determining vector competences and host preferences of the different potential vectors [9].

Given the borderless nature of RVFV risk, risk management strategies will need to be pan-regional and based on cooperation through the exchange of information and the pooling of resources [123,124,126].

## Surveillance of points of entry according to International Health Regulations

The International Health Regulations (IHRs) were revised in 2005 and explicitly call for vector surveillance and control. In 2016, WHO published a handbook providing guidance in implementing vector surveillance and control within this framework [131]. With specific regard to the definition of entomological surveillance, particular emphasis is given to establishing global baseline conditions for points of entry (their natural and urban environments and their surroundings, the local entomological situation and epidemiological context) for implementing targeted and proportionate surveillance.

## Conclusion

Over recent decades, mosquito-borne viruses, including CHIKV, DENV, RVFV, WNV, YFV, and ZIKV, have emerged or reappeared, posing a threat to global health. Efficient surveillance systems are, therefore, of the utmost importance. In light of the various issues discussed previously, surveillance systems must be defined according to the pathogen being targeted and adapted to the epidemiological and entomological contexts and existing resources. Surveillance systems should be strengthened in accordance with the One Health framework so that the different components involved in the introduction and spread of VBDs (vector–pathogen–hosts) can be addressed globally. The definition of any surveillance system should be based on assessment of the national and local situation in order to identify priority areas for multisectoral efforts (e.g., entomology, virology, and human and veterinary public health). A comprehensive capacity-building strategy to confront VBDs must focus on the main competences needed to deal with this risk, but there must also be awareness of and a capacity to develop tools to foster collaboration. Integrated public health policies call for the interoperability of surveillance systems for the purposes of sharing information and carrying out joint data analysis.

International One Health networks, such as MediLabSecure, are important tools for reinforcing preparedness and response to global health threats, as they (1) contribute to the sharing and dissemination of good practises and experiences among countries; (2) foster collaboration between different sectors, thereby overcoming disciplinary barriers; (3) provide specialised training to improve the capacities of the sectors involved; and (4) are able act as a force to influence public health priorities at both global and local levels by instigating interdisciplinary activities that extend traditional fields of competence.

**Key learning points**

- The prevention and management of mosquito-borne viral diseases must be addressed in the prism of the One Health strategy, which holds entomological surveillance to be an integral part of policy.
- Any surveillance system should be defined on the basis of assessments of national and local situations to identify priority areas for multisectoral efforts.
- Entomological surveillance systems should be designed according to the entomological and epidemiological context and well-defined objectives in order to develop a tailored, graduated response.
- Robust surveillance systems are needed to address the globalisation of emerging arboviruses. This calls for increased capacities and stronger collaborations between sectors at both the local and regional levels.
- The development of multidisciplinary networks, involving both academics and public authorities, provides a means to address these health challenges by promoting good practises in surveillance (design of surveillance systems, data collection, etc.) and the sharing of knowledge and information.

**Top five papers**

1. Chevalier V, Lecollinet S, Durand B. West Nile virus in Europe: a comparison of surveillance system designs in a changing epidemiological context. Vector Borne Zoonotic Dis. 2011 Aug;11(8):1085–91. 10.1089/vbz.2010.0234. Epub 2011 May 6.
2. Dente MG, Riccardo F, Nacca G, Ranghiasci A, Escadafal C, Gaayeb L, Jiménez-Clavero MA, Manuguerra JC, Picard M, Fernández-Pinero J, Pérez-Ramírez E, Robert V, Victoir K, Declich S. Strengthening Preparedness for Arbovirus Infections in Mediterranean and Black Sea Countries: A Conceptual Framework to Assess Integrated Surveillance in the Context of the One Health Strategy. Int J Environ Res Public Health. 2018 Mar 10;15(3):E489. 10.3390/ijerph15030489.
3. Gossner CM, Marrama L, Carson M, Allerberger F, Calistri P, Dilaveris D, Lecollinet S, Morgan D, Nowotny N, Paty MC, Pervanidou D, Rizzo C, Roberts H, Schmoll F, Van Bortel W, Gervelmeyer A. West Nile virus surveillance in Europe: moving towards an integrated animal-human-vector approach. Euro Surveill. 2017 May 4;22(18): 30526. 10.2807/1560-7917.ES.2017.22.18.30526.
4. Schaffner F, Mathis A. Dengue and dengue vectors in the WHO European region: past, present, and scenarios for the future. Lancet Infect Dis. 2014 Dec;14(12):1271–80. 10.1016/S1473-3099(14):70834–5. Epub 2014 Aug 26.
5. Stärk KD, Regula G, Hernandez J, Knopf L, Fuchs K, Morris RS, Davies P. Concepts for risk-based surveillance in the field of veterinary medicine and veterinary public health: review of current approaches. BMC Health Serv Res. 2006 Feb 28;6:20.

## Supporting information

S1 Table The 19 member laboratories of the medical and veterinary entomology MediLabSecure network who took part in the online survey. (XLSX)
